# Editorial: From Genes to Species: Novel Insights from Metagenomics

**DOI:** 10.3389/fmicb.2016.01181

**Published:** 2016-08-03

**Authors:** Eamonn P. Culligan, Roy D. Sleator

**Affiliations:** Department of Biological Sciences, Cork Institute of TechnologyCork, Ireland

**Keywords:** metagenomics, functional metagenomics, metatranscriptomics, next generation sequencing, microbiome

The majority of microbes in many environments are considered “as yet uncultured” and were traditionally considered inaccessible for study through the microbiological gold standard of pure culture. The emergence of metagenomic approaches has allowed researchers to access and study these microbes in a culture-independent manner through DNA sequencing and functional expression of metagenomic DNA in a heterologous host. Metagenomics has revealed an extraordinary degree of diversity and novelty, not only among microbial communities themselves, but also within the genomes of these microbes. Metagenomic analysis can involve sequence-based or functional approaches (or a combination of both). The continuous improvements to DNA sequencing technologies coupled with dramatic reductions in cost have allowed the field of metagenomics to grow at a rapid rate. Many novel insights on microbial community composition, structure, and functional capacity have been gained from sequence-based metagenomics. Functional metagenomics has been utilized, with much success, to identify many novel genes, proteins, and secondary metabolites such as antibiotics with industrial, biotechnological, pharmaceutical, and medical relevance. Future improvements and developments in sequencing technologies, expression vectors, alternative host systems, and novel screening assays will help advance the field further by revealing novel taxonomic and genetic diversity. This Research Topic aims to showcase the utility of metagenomics to gain insights on the microbial and genomic diversity in different environments by revealing the breadth of novelty that was in the past, largely untapped. This Research Topic comprises 19 submissions from experts in the field and covers a broad range of themes and article types (Review, Methods, Perspective, Opinion, and Original Research articles). We have broadly grouped the articles under four themes; functional metagenomics, targeted metagenomics, sequence-based metagenomics, and host-associated.

We begin with a number of articles focusing on functional metagenomics. A review by Coughlan et al. gives an overview of metagenomics and focuses on the utility of functional metagenomics for the discovery of proteins and antimicrobial compounds with relevance to the food and pharmaceutical industries. Continuing this theme Uchiyama et al. report the discovery of a glucose-tolerant β-glucosidase from screening ~10,000 clones from a metagenomic library created from Kusaya gravy (a traditional Japanese fermentate made from fish). β-glucosidases are often sensitive to glucose inhibition, therefore glucose-tolerant variants are desirable to improve enzymatic efficiency. Mirete et al. also used a functional metagenomic approach to identify novel salt tolerance genes from brine and rhizosphere-associated communities in a hypersaline saltern. A number of the genes had not previously been known to play a role in salt tolerance. This approach demonstrates one of the main advantages of functional metagenomics; assigning function to unknown genes or new functions to annotated genes.

As with any technology, there are advantages and disadvantages. In their Perspective article, Lam et al. present the main challenges and potential solutions associated with functional metagenomics. Biases may be introduced at different stages of the process, from DNA extraction, library construction, cloning, and choice of expression vector and heterologous host. The authors discuss advances to improve each step and provide helpful comments based on their own considerable experience. They also present data, which suggests cloning bias is occurring at the level of individual operational taxonomic units (OTUs). Finally, it is suggested that moving beyond *Escherichia coli* as a cloning host will increase the diversity of hits from functional screens. An additional issue associated with metagenomics it that there is still a dearth of functional information for a large proportion of protein families; a problem which is increasing due to the enormous amounts of sequencing data that continues to be generated and deposited in databases. Ufarté et al. review sequence-based and activity screening approaches in metagenomics to assign functions to novel genes. The authors also discuss recent developments in microfluidic approaches for ultra-high-throughput screening, where up to 1 million clones can be assessed in a single day.

On a similar theme, Suenaga discusses the role of “targeted” metagenomics in compiling specific groups of enzymes to study their adaptive evolution, and echo the importance of the microfluidics approach mentioned above, as well as technologies such as cell compartmentalisation, flow cytometry, and fluorescent cell sorting in the future for high-throughput screening. Trindade et al. reviews how targeted metagenomics may be used to identify natural products from marine organisms and microbes, which have the potential to treat human disease. The authors explain why functional screening approaches have been largely unsuccessful in this regard. However, using targeted metagenomic approaches, guided by well-known structural and functional characteristics of natural products, a number of clinically relevant compounds have been successfully isolated; including several potent anti-cancer and anti-fungal compounds such as, bryostatins, patellazoles, polytheonamides, ecteinascidin 743, pederin, psymberin, and calyculin A. Dziewit et al. describe a targeted approach to detect methanogenic archaea. Methanogenic archaea are important community members of many diverse environments including peatlands, freshwater sediments, and the intestinal tract of animals and humans. Many members have proved difficult to culture and previous studies have relied on metagenomic, 16S rDNA, and *mcrA* gene sequencing. The authors present a methods paper detailing the development of a number of sets of degenerate primers for methanogenic archaea based on the *mcrB, mcrG, mtbA*, and *mtbB* genes, which are involved the process of methanogenesis. These novel molecular markers will provide additional information on the biology, diversity, and phylogenetic relationships of these organisms.

Sequence-based metagenomics can provide unprecedented information on composition, diversity, and functional capacity of microbial communities. One of the main challenges associated with sequence-based metagenomics is *de novo* assembly of reads following sequencing. Howe et al. outline some of the main issues with such assemblies. The authors also include a unique iPython notebook tutorial that allows readers to follow the steps of this process and execute assembly of a mock metagenome.

Wemheuer et al. assessed the effect of phytoplankton *Phaeocystis globosa* algal bloom on microbial communities in the North Sea, using metagenomic, and metatranscriptomic approaches. Changes in community composition were identified inside the bloom in comparison to outside the bloom, most likely due to changing nutrient availabilities during algal bloom growth. Indeed, metatranscriptomic data revealed changes in gene expression in response to the bloom. Genes for incorporation of leucine and isoleucine were significantly upregulated and many genes encoding transposases were overexpressed inside the bloom. It is suggested that genome rearrangement via expression of transposases enables increased stress resistance and enhanced adaptation to changing environmental conditions.

Using a similar metagenomic and metatranscriptomic approach, He et al. investigated microbial sulfur cycling and carbon and nitrogen metabolism in a hydrothermal chimney. The genes identified were used to unravel potential pathways for sulfur and carbon metabolism, which play an important role for survival in this environment. Furthermore, γ-proteobacteria, and ε-proteobacteria are proposed as community members capable of denitrification, using electrons generated from oxidation of reduced sulfur. Bargiela et al. report a bioinformatic analysis of a previously published metagenomic dataset to identify genes enriched in a crude-oil-contaminated marine environment. Specifically, genes enriched following ammonium and uric acid (bio-stimulants) treatment were identified. Differences in taxonomic composition, presence of genes and metabolic pathway constituents and biodegradation were noted following bio-stimulant treatment. Both bio-stimulants appeared to increase the capacity for microbial degradation of crude oil.

Rosario et al. present research on the area of viral metagenomics. Twenty-seven novel CRESS-DNA (circular Rep-encoding ssDNA) viruses were identified and sequenced from marine invertebrates, some of which may represent a novel family. Intrinsically disordered regions (IDRs) within proteins were also investigated. IDRs lack rigid structure and allow the protein to exist in different states, which may allow multi-functionality in such proteins. Different IDRs are commonly found in proteins encoded by CRESS-DNA viruses and may be useful to characterize divergent structural proteins, though at present the importance of the different IDRs remains to be confirmed.

Bao et al. used strand-specific metatranscriptomics in a novel way to identify anti-sense transcription among members of the human gut microbiota. Anti-sense RNAs are encoded on the opposite strand of DNA from the mRNA transcript and may have important regulatory functions in gene expression. Most of the species tested displayed anti-sense transcription (ranged from 0 to 38.5% for protein coding genes between different species). Interestingly, the functional category of genes most over-represented with anti-sense transcription included prophage-associated and transposon genes.

Metagenomic approaches have provided a wealth of information about the microbes on and in the human body (microbiota) and their potential role in human health and disease. Belizario and Napolitano review current information on a number of human microbiomes (gut, oral, skin, placental), and discuss how targeting and mining the microbiota is opening a new area of microbiome-based therapeutics. For example, the use of probiotics and prebiotics, phage therapy and CRISPR technology are exciting areas of research, while faecal microbiota transplantation (FMT) has shown promising results for the treatment of *Clostridium difficile* infection (CDI). Kinumaki et al. use metagenomic sequencing to profile the gut microbiota of patients with Kawasaki disease (KD), an acute childhood illness characterized by vascular inflammation, which is a leading cause of acquired heart disease. The precise cause of KD is unknown, but a possible microbial influence has been suggested to play a role in its pathogenesis. Metagenomic sequencing revealed differences in gut microbiota composition between KD patients during acute and non-acute phases of the disease. In particular, a number of species from the genus *Streptococcus* were significantly increased during the acute phase of KD. The authors suggest that species of *Streptococcus* may play a role in KD pathogenesis, but more research is required to conclusively demonstrate a causal link.

Brito and Alm, review strain-level tracking of microbes using metagenomics. The authors state that transmission has primarily focused on pathogenic organisms, but very little is known about transmission of commensal species. With significant emerging evidence for the roles that commensal microbes play in human health and disease, the ability to track, and differentiate microbes at the strain level is important. Metagenomic sequencing provides advantages over 16S rDNA sequencing in this regard for example, and long-read sequencing (e.g., Oxford Nanopore's MinION) and proximity ligation (enables detection of protein-protein and protein-DNA interactions, as well as post-translational modifications) may help improve this in the future. The ability to track strain-level transmission will be key to monitor live microbial therapeutics and the biological containment of engineered microorganisms, while longitudinal studies could reveal how transmission affects daily or intermittent changes to the microbiota.

Voss et al. propose the “pawnobiome” as a “subset of the microbiome that is purposefully managed for manipulation of the host phenotype, which includes individual microbes named pawnobes.” Different from the hologenome theory of evolution, where the unit of selection is the holobiont (i.e., both the host and its associated microbiota); the pawnobiome can evolve independently and faster than the host and is not wholly reliant on host survival. It is also proposed that the pawnobiome can affect host phenotype and can be independently/artificially selected; thus having implications for health and disease, biotechnology, and evolutionary biology.

Finally, Simm et al. present an analysis of the core- and pan-genome of cyanobacteria. Using 58 sequenced cyanobacterial genomes, the authors identify 559 genes that define the core-genome. Furthermore, 3 genes specific to thermophilic cyanobacteria and 57 genes specific to heterocyst-forming cyanobacteria were also defined. Additionally, outer membrane β-barrel proteins were investigated. It was found that most of these proteins are not globally conserved and exhibit strain specificity, indicating cyanobacteria have evolved individual strategies for environmental adaptation and interaction.

Overall, this Research Topic showcases a broad range of articles which illustrate the utility of both sequence-based and functional metagenomic approaches to investigate what were once inaccessible and undiscovered areas of microbial genomics, physiology, evolution, and ecology. Future advances in metagenomic research and technology will undoubtedly reveal further novelty and diversity from genes to species and beyond.

## Author contributions

EC and RS co-edited the Research Topic. Both authors wrote, edited, and approved the final version of the Editorial.

### Conflict of interest statement

The authors declare that the research was conducted in the absence of any commercial or financial relationships that could be construed as a potential conflict of interest.

